# Barriers and Facilitating Factors for Implementation of Genetic Services: A Public Health Perspective

**DOI:** 10.3389/fpubh.2017.00195

**Published:** 2017-08-04

**Authors:** Martina C. Cornel, Carla G. van El

**Affiliations:** ^1^Department of Clinical Genetics, Amsterdam Public Health Research Institute, VU University Medical Center, Amsterdam, Netherlands

**Keywords:** public health genomics, BRCA1, BRCA2, hereditary colon cancer, cascade testing, service development

## Abstract

More than 15 years after the publication of the sequence of the human genome, the resulting changes in health care have been modest. At the same time, some promising examples in genetic services become visible, which contribute to the prevention of chronic disease such as cancer. These are discussed to identify barriers and facilitating factors for the implementation of genetic services. Examples from oncogenetics illustrate a high risk of serious disease where prevention is possible, especially in relatives. Some 5% of breast cancers and colorectal cancers are attributable to an inherited predisposition. These cancers occur at a relatively young age. DNA testing of relatives of affected patients may facilitate primary and secondary prevention. Training of non-genetic health care workers and health technology assessment are needed, as is translational research in terms of bringing genomics to health care practice while monitoring and evaluating. Stratified screening programs could include cascade screening and risk assessment based on family history. New roles and responsibilities will emerge. A clear assessment of the values implied is needed allowing to balance the pros and cons of interventions to further the responsible innovation of genetic services.

## Introduction

After the first draft of the sequence of the human genome had been published in the year 2000, expectations were that this would revolutionize diagnosis, prevention, and treatment of most, if not all, human diseases; however, a decade later, changes in health care were modest (1). The price of genome information has decreased very fast after the year 2000, especially when “next-generation” sequencing was introduced around 2007 (www.genome.gov/sequencingcosts). In 2016, the cost of a whole genome sequence was close to 1,000$. However, this does not include the analysis of the enormous amount of data. Those who were disappointed about the modest consequences for clinical medicine should remember the First Law of Technology: we invariably overestimate the short-term impacts of new technologies and underestimate their longer term effects (1).

This article reflects a presentation given at the European Public Health Conference in Vienna, November 11, 2016, at the Round table “Bridging the gap between knowledge and practice in public health genomics,” organized by participants of the PRECeDI project funded by the European Commission (Personalized PREvention of Chronic Diseases). The aim of this study is to discuss how public health can benefit from promising examples of genetic testing, such as in cases of hereditary forms of breast and colorectal cancer, and what barriers and facilitating factors should be addressed for a successful implementation.

**Figure 1:**
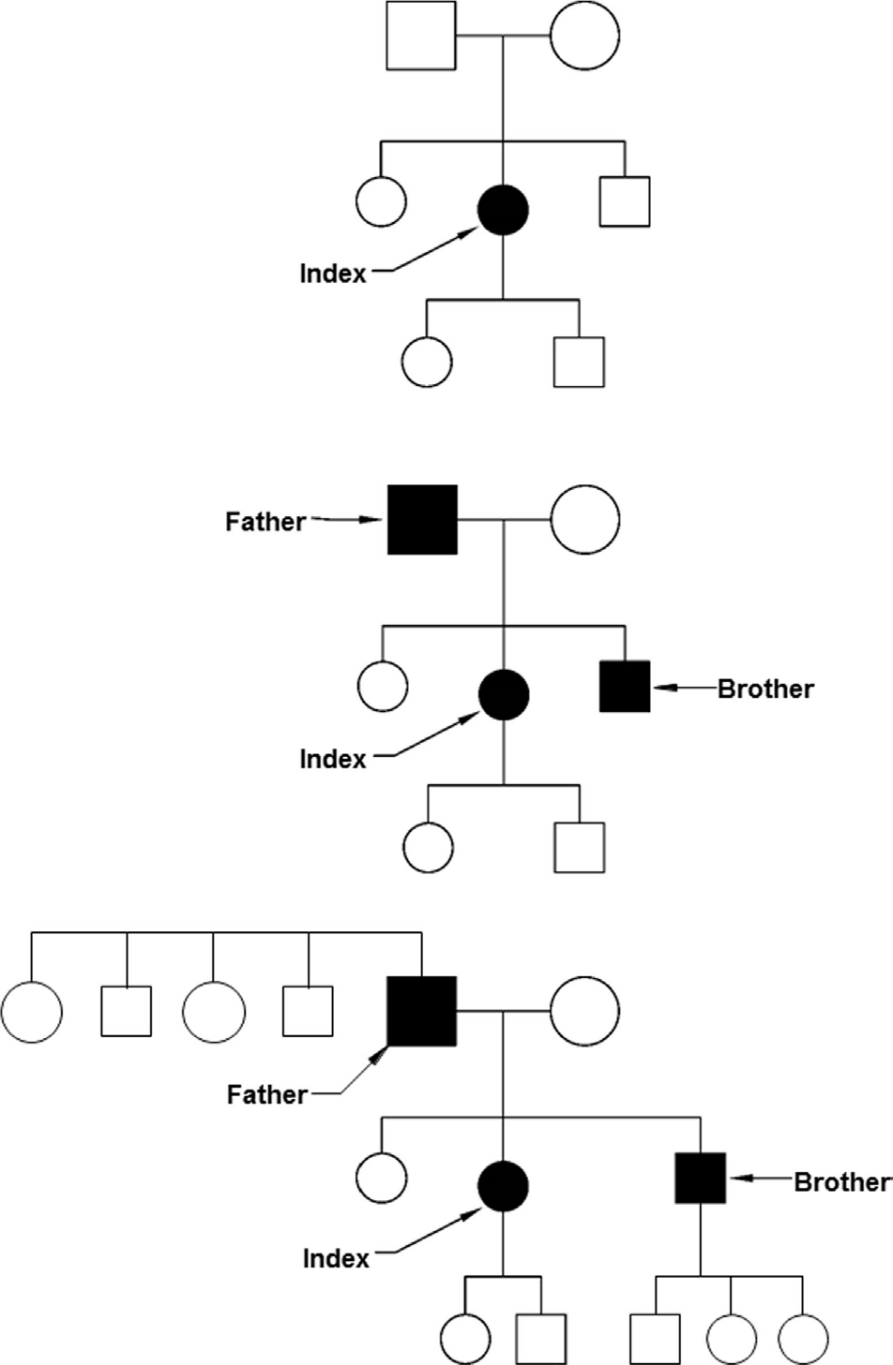
Cascade screening. Top: after the identification of an index patient, first-degree relatives are invited to be tested (parents, brothers, sisters, and children). Middle: some of the first-degree relatives may be diagnosed before they have symptoms. Below: also the first-degree relatives of the presymptomatically identified patients can be invited, including brothers and sisters of the father and the children of the son.

If the limited resources (both staff and funding) available in clinical genetics require a focus on priorities, considerations of medical benefit, health need, and costs apply (2). This is similar for many other fields in health care, where evidence of clinical benefit and benefit of information for important life decisions are criteria for prioritization. However, in the case of clinical genetics, also the benefit for other people apart from the person tested and the patient-specific likelihood of being affected by the condition tested for play a role. Identifying one person at risk of a serious but preventable complication of a hereditary disease with 25–50% risk for relatives makes testing of family members and preventative treatment possible. From a public health perspective, an approach to use the family structure to first identify those at the highest risk and thus most likely to profit from evidence-based interventions is attractive (Figure 1). While screening programs often invite specific age groups (e.g., newborns, women ≥50 years), a cascade screening would invite family members. Adoption of cascade services should yield substantial quality of life and survival gains (3). After the diagnosis in an index patient, an invitation would be sent to parents, brothers and sisters, and children. If any of them is also diagnosed with the condition, a next circle of first-degree relatives will be invited. This systematic approach is very effective in autosomal dominant conditions. Examples are *BRCA*-related breast cancer and colorectal cancer in Lynch syndrome families as monogenic subtypes of common cancers (4). A risk of 50% to carry the mutation applies for first-degree relatives in these conditions.

Ethical and legal issues have been discussed in relation to cascade screening strategies, especially the active approach of family members. To what extent is there a right “not to know” vs. a “duty to warn” (5, 6)? Newson and Humphries (5) argue that relatives’ autonomy will be best respected by their knowing that a risk is present and receiving assistance in coming to a decision about whether to undergo testing. De Wert (6) argues that critics tend to ignore that relatives may have the “right to know,” conditional upon the preventive value of the information. An ethical view that focuses exclusively on relatives’ right not to know does not do justice to the (possible) relatives’ health interests. In a broader sense, in the last decade, an ethical duty is recognized to return individual genetic research results subject to the existence of proof of validity, significance, and benefit (7), while balancing advantages against the right not to know.

## BRCA

BRCA is an abbreviation for BReast CAncer susceptibility gene. Mutations in the two genes *BRCA1* and *BRCA2* predispose for breast cancer. Many countries have breast cancer screening programs where mammography is offered to women above a certain age limit, for instance, starting at 50 years in the Netherlands. Women with a mutation in the *BRCA1*-gene or *BRCA2*-gene may develop cancer at a younger age. Their risk to develop breast cancer before 80 years of age is 66–67% (8). At age 50, already 20–30% of the mutation carriers developed breast cancer. Furthermore, they have an increased risk of ovarian cancer, 45% for *BRCA1* and 12% for *BRCA2* mutation carriers before 80 years of age (8). Apart from surveillance at a younger age, by mammography or MRI, also risk-reducing mastectomy and risk-reducing salpingo-oophorectomy are options for the primary prevention of breast and ovarian cancers. For relatives of cancer patients in whom a mutation has been found, it is possible to use these surveillance and prevention possibilities. For first-degree relatives (such as sister and daughters) of an index patient, the *a priori* risk to carry the familial mutation is 50%. In the media, this possibility for prevention attracted attention when Angelina Jolie, an American actress, told about her choice to be proactive and minimize the risk as much as she could, so that her children would not need to fear that they would lose her to breast cancer.

## Hereditary Colorectal Cancer

Around 5% of all colorectal cancers are associated with an inherited predisposition. Among persons with mutations in the mismatch repair genes, the most frequent subgroup, lifetime colorectal cancer risk is 30–70% (9). Colonoscopy is advised every 1–2 years, starting at age 20–25. In classic familial adenomatous polyposis, accounting for <1% of colorectal cancer cases, risks are even higher (lifetime risk >90%), and surveillance may start at the age of 10. Colon cancer screening programs in many countries start at a much higher age, too late for the mutation carriers. Trials with aspirin chemoprevention are ongoing, which would provide a cheap possibility for primary prevention (10). The testing of relatives of persons recognized with inherited forms of colon cancer would become even more relevant to increase survival rates if not only colonoscopy surveillance but also chemoprevention would prove to be effective.

## Barriers

While some applications of genetics in public health become available, there are barriers for their implementation as well. The first we will discuss is the lack of genetic knowledge and competences. If physicians working in public health, or general physicians who are non-genetic experts, are to take a role in the development and delivery of genetic services and in identifying family members at risk, a lack of genetic knowledge relevant for every day care is a major problem (11). Curricula may focus on scientific aspects of human genetics, overlooking some other competences, such as taking a family history, drawing a family tree, and knowing when and how to refer to a clinical genetic center. Referral criteria are sometimes phrased as “one first degree relative with cancer under the age of …,” or “two second degree relatives ….” Knowing who are first-, second-, and third-degree relatives is thus genetic knowledge needed for every day care. Genetics educational interventions are little studied, and existing studies often focus on knowledge, while changes in practice are also needed to be effective in genetic risk assessment and appropriate management of patients (12). Starting from a needs assessment in primary care it turned out to be possible to develop effective training to identify patients at risk for hereditary cancers (13). For other fields of (genetic) health care, similar studies are needed, as are regular updates when new services become available and/or new roles are defined.

A second barrier for implementation is the lack of health technology assessment for the application of genetics. As many genetic conditions are rare, a general issue is that alternative designs may be needed for the evaluation of scientific evidence in rare diseases (14). Randomized clinical trials may not be appropriate for persons with rare conditions often caused by different mutations that may have different risk profiles. Both the evaluation of a specific treatment for a rare gene variant and the evaluation of the clinical utility of testing may demand new study designs. Observational studies and biological insights will be considered low grades of evidence. If personalized medicine is the future, analytical considerations such as Bayesian analysis and use of biomarkers as surrogate outcomes may be considered (14).

A third barrier is the lack of translational research in terms of translation “from bench to bedside” unlike translational research “from mice to man.” No more than 3% of published genomics research focuses on research beyond the first phase of translation (15). The higher phases of translation include assessment of the value of a genomic application for health practice, evidence-based guidelines delivery, dissemination and diffusion research, and evaluation of health outcomes of a genomic application in practice.

A fourth barrier relates to the slow pace of translation, which in turn led to commercial offers direct-to-consumers of tests, often with low predictive value. This may undermine trust of citizens and health care professionals.

Furthermore, the lack of availability of resources and access to these resources, including laboratories and personnel, may limit the application of genetics. Ethical issues and lack of approval of innovative testing strategies may also be barriers.

## Facilitating Factors

The cases of BRCA testing and hereditary colorectal cancer can help to recognize which facilitating factors played a role in these promising examples. In terms of the innovation curve, these examples moved beyond the phase of early adopters. Serious diseases where positive testing results would have a high positive predictive value and where interventions are available are the first for which genetic services should be implemented. In terms of health technology assessment, they have added value to existing healthcare. Interventions can be both at the level of secondary prevention (colonoscopy to remove polyps and thus prevent colorectal cancer) or primary prevention (chemoprevention by aspirin). Since the cancers prevented in these inherited cancer syndromes tend to occur at a relatively young age, the number of (quality adjusted) life years saved is relatively high.

Public awareness is another facilitating factor. It can be increased by a famous person such as Angelina Jolie who found herself in the position of being at risk of hereditary breast and ovarian cancers.

Furthermore, initiatives to train relevant health care professionals including public health genetics can open doors. If already some ground work has been done, the next steps may be easier.

## Public Health Genetics: Work to be Done

A first field where public health and genetics meet is cancer screening. Cascade screening as discussed above could be developed in a program, but risk assessment in a broader sense is conceivable. Would it be possible to develop a breast cancer screening program stratified according to risk profile (16)? As soon as the 10-year breast cancer risk would be ≥2.4%,—the current threshold for breast cancer screening in the UK at age 47—a woman could start mammography. For a low-risk group without family history, this might not be before 80 years, while for the highest risk, it would be before the age of 35 years. Apart from the development of polygenic risk models, it would be necessary to identify the optimal service delivery mechanisms, including (but not limited to) cost-effectiveness and cost–benefit evaluations of alternative implementation plans, as well as monitoring and surveillance.

In colon cancer screening, many countries are now integrating universal colorectal cancer tumor screening in health care to identify families at higher risk of colorectal cancer due to Lynch syndrome (17). An important question is how to organize this and how to define the roles for different professionals. A clear division of responsibilities is crucial to efficiently and effectively form or change the structure of the new practice (4). To attune new roles and responsibilities and manage the transition, public health institutes could play an important role.

A second field where public health and genetics meet is genetic education. If the public at large is to profit from recent developments in genetics, any physician or health worker should be able to answer questions about genetics relevant for daily practice. The relatively small number of registered clinical geneticists will not be able to teach all physicians in face-to-face training sessions. Smart solutions such as online modules, webinars, and websites are needed (13, 18). The relevance of Public Health Genomics education among public health specialists has been recently acknowledged by the Association of Schools of Public Health in the European Region, and 15 schools have at least 1 dedicated course in place (19). However, further harmonization of the training programs of schools in public health at EU level is needed.

A third field where public health and genetics meet is the study of the cost-effectiveness or health technology assessment in a broader sense. This would include the assessment of values such as the extent to which patient perspectives have been taken into account, life years gained, quality adjusted life years, and percentage of cases identified. We need to discern which applications are hype vs. hope. If limited resources are available, which need prioritization? Both naïve believers and individuals who are resistant to change may be encountered as barriers for implementation of genetic services. To further the responsible innovation of genetic services a clear assessment of the relevant indicators and the implied values is needed allowing to balance the pros and cons of interventions (20). A clear assessment of the values implied allowing to balance the pros and cons of interventions is needed to further the responsible innovation of genetic services.

To conclude, in cancer screening, genetic education, and (economic) evaluations, much work needs to be done for both the public health and genetics communities to address barriers and make use of promising developments to further a successful and responsible implementation of genetic services in public health.

## Author Contributions

Both MC and CE were involved in conception and design of the work for the PRECeDI project on integrating genetics in the prevention of chronic diseases. MC drafted the presentation and manuscript. CE revised it critically. Both authors approve the final version and accountable for all aspects of the work.

## Conflict of Interest Statement

The authors declare that the research was conducted in the absence of any commercial or financial relationships that could be construed as a potential conflict of interest.
